# Polarisers in the focal domain: Theoretical model and experimental validation

**DOI:** 10.1038/srep42122

**Published:** 2017-02-13

**Authors:** Rosario Martínez-Herrero, David Maluenda, Ignasi Juvells, Artur Carnicer

**Affiliations:** 1 Universidad Complutense de Madrid, Facultad de Ciencias Físicas, Departamento de Óptica, Ciudad Universitaria, 28040 Madrid, Spain; 2 Universitat de Barcelona (UB), Facultat de Física, Departament de Física Aplicada, Martí i Franquès 1, 08028 Barcelona, Spain

## Abstract

Polarisers are one of the most widely used devices in optical set-ups. They are commonly used with paraxial beams that propagate in the normal direction of the polariser plane. Nevertheless, the conventional projection character of these devices may change when the beam impinges a polariser with a certain angle of incidence. This effect is more noticeable if polarisers are used in optical systems with a high numerical aperture, because multiple angles of incidence have to be taken into account. Moreover, the non-transverse character of highly focused beams makes the problem more complex and strictly speaking, the Malus’ law does not apply. In this paper we develop a theoretical framework to explain how ideal polarisers affect the behavior of highly focused fields. In this model, the polarisers are considered as birefringent plates, and the vector behaviour of focused fields is described using the plane-wave angular spectrum approach. Experiments involving focused fields were conducted to verify the theoretical model and a satisfactory agreement between theoretical and experimental results was found.

Polarisers are devices present in almost all optical set-ups. They are used to project the electric field of the beam onto a direction given by the orientation of the polariser axis. In most optical systems beams propagate along the optical axis; in this case the mathematical description of the effect of the polariser is simple. Nevertheless, a more accurate model has to be used if the beam impinges the polariser with a certain angle with respect to the normal direction of the plane of the polariser. The problem becomes more complex when dealing with highly focused fields since multiple angles of incidence have to be taken into account. Moreover, the electric field is not transverse to the direction of propagation and the contribution of the longitudinal component plays a role on how the field interacts with a polariser^1^,^2^,^3^. In the last years, beam shaping and highly focused beams attracted great interest in multiple research areas such as electron acceleration, nonlinear optics, optical tweezers, optical security, etcetera^4^,^5^,^6^,^7^,^8^,^9^,^10^,^11^,^12^,^13^,^14^,^15^,^16^,^17^. For this reason, in our opinion it is required to develop a theoretical framework that explains how ideal polarisers affect the behaviour of beams in the focal domain.

In this work, polarisers are considered to be optically equivalent to thin uniaxial anisotropic plates. The transmission of light through birefringent layers has been analysed using different approaches. For instance, the 4 × 4 matrix method provides exact solutions^18^,^19^,^20^. However, the simpler 2 × 2 matrix technique offers accurate results if the effect of multiple internal reflections can be neglected^21^,^22^,^23^,^24^,^25^. This approximation is valid for most practical birefringent devices because they are relatively thick. For example, this model was used to describe obliquely-illuminated twisted nematic liquid-crystal displays^23^,^24^,^25^. The objective of this paper is to analyse the effect of an ideal polariser on a highly focused field. In particular, we develop a theoretical approach for polarisers in the focal domain using the angular representation of the field and the 2 × 2 model for uniaxial media. Theoretical results are supported by experiments. To the best of our knowledge this is the first experimental report on polarisers and highly focused beams.

The paper is organized as follows: First, we develop a theoretical model for polarisers based on uniaxial anisotropic media. This approach provides an explanation on how highly focused fields interacts with linear polarisers in the focal area. Numerical and experimental results are presented and discussed. Finally, we review the physical principles of birefringent media and how are they related with polarisers. A detailed explanation of the experimental set-ups is provided.

## Results

### Behaviour of a linear polariser on a highly focused field

**Figure 1:**
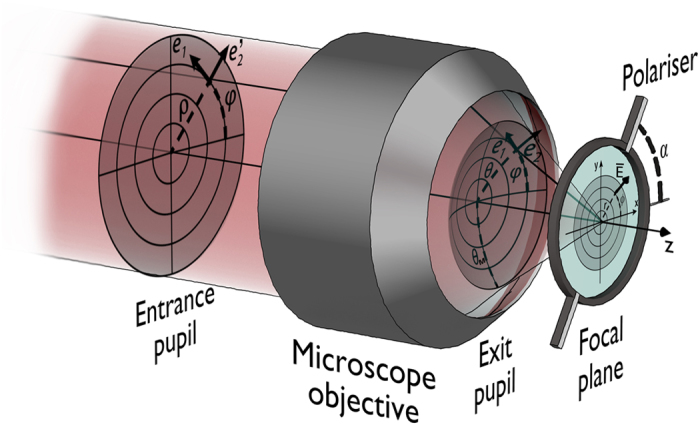
Coordinate system and geometrical magnitudes.

The electric field distribution in the focal region of a high numerical aperture optical system is given by the Richards-Wolf integral^26^


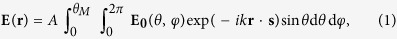


where A is a constant related to the focal length and the wavelength, *k* = 2*π*/*λ* is the wave number, *θ*_*M*_ is the semi-aperture angle, **r** = (*r, ϕ, z*) denotes the polar coordinates at the focal area, *θ* and *φ* are the coordinates at the Gaussian sphere and 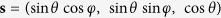 is the wave-front vector. The numerical aperture (NA) and *θ*_*M*_ are related by means of NA = sin *θ*_*M*_ (see Fig. 1 for details). **E**_0_ is the so-called vectorial angular spectrum, namely


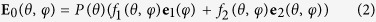


where


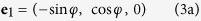



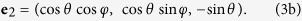


Note that **e**_2_ is contained in the plane of incidence and **e**_1_ and **e**_2_ are orthogonal, i.e. **e**_1_ ⋅ **e**_2_ = 0; symbol ⋅ denotes scalar product. For an isoplanatic system, the apodization function becomes 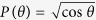; *f*_1_(*θ, φ*) and *f*_2_(*θ, φ*) are, respectively, the azimuthal and radial transverse components of the incident paraxial field.

Let us now consider an ideal linear polariser characterized by polarization angle *α* (see Fig. 1). According to the model of polarisers as uniaxial anisotropic media^21^,^22^, the contribution of each plane wave of the angular spectrum is described by


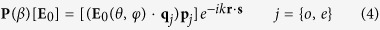


where sub-indices *o* and *e* stand for O-type and E-type polarisers respectively. The direction of the optical axis (*c*-axis) is described by 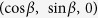 where *α* and *β* are related by *α* = *β* + *π*/2 for O-type polarisers and *α* = *β* for E-type polarisers. Vectors **q**_*o*_, **q**_*e*_, **p**_*o*_ and **p**_*e*_ are given by


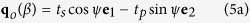



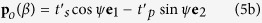



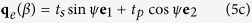



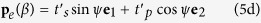


where


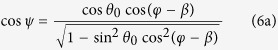



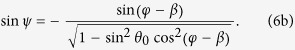


Finally, the Fresnel transmission coefficients read


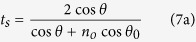



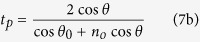



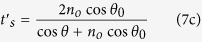



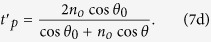


More information about this model can be found in the Methods section. Note that *t*_*s*_ and *t*_*p*_ are evaluated at the first surface, coefficients *t*′_*s*_ and *t*′_*p*_ are related to the second surface, *θ*_0_ is the refraction angle that fulfils 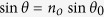, and *n*_*o*_ is the ordinary refractive index. Moreover, under the small-birefringence approximation 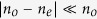, both ordinary and extraordinary waves propagates in the same direction (*n*_*e*_ is the extraordinary refractive index)^21^,^22^.

The electric field of the focused beam after the polariser **E**_*p*_(**r**, *β*) is given by combining Eqs (1) and (4):


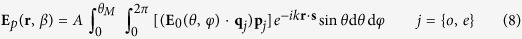


From the previous equation follows that the features of the field after the polariser depend on the polarization state of the vector angular spectrum **E**_0_(*θ, ϕ*), its topological charge and the numerical aperture of the objective lens. Moreover, Eq. (8) clearly states that **E**_*p*_(*r, β*) is non-uniformly polarised and presents a longitudinal component even in the case that the field that crosses the polariser is purely transverse. For instance, according to Eq. (2) an azimuthally polarised incident beam (with *f*_2_ = 0) displays a non-zero longitudinal component after the polariser.

**Figure 2:**
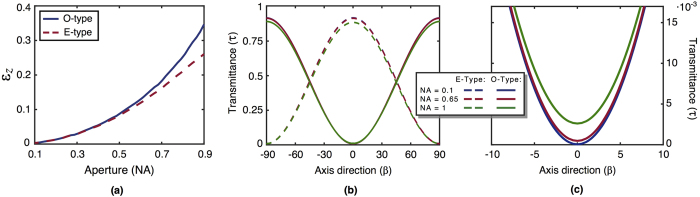
(**a**) Ratio between the total power of the longitudinal component and the entire field, *ε*_*z*_(NA); (**b**) Ratio between the energy transmitted and the energy of the incident field as a function of the direction of polarization, transmittance *τ(β*); (**c**) zoom of the minimum of transmittance *τ(β*) for O-type polarisers.

In order to provide more insight about how ideal polarisers modify the behaviour of a field in the focal area, two parameters are introduced: (i) the ratio *ε*_*z*_ between the total power of the longitudinal component and the entire field and (ii) the transmittance (a.k.a. gain) *τ*, defined as the ratio between the energy transmitted and the energy of the incident field^27^. Figure 2a shows the behaviour of *ε*_*z*_ as a function of NA for the O- and E- cases. The input beam is linearly polarised orthogonal to the polariser axis. In this paper we used the following values for the calculations involving refractive indexes: *n*_*o*_ = 1.55 and *n*_*e*_ = 1.556. Note that even for low NA values, the transmitted longitudinal component is non-negligible. Figure 2b show the behaviour of *τ* as a function of polariser angle *β*. It is apparent that maximum or minimum transmittances are reached when ***E***_0_ is parallel or perpendicular to **q**_*e*_ or **q**_*o*_, respectively. Figure 2c is a zoom of Fig. 2b around *β* = 0°. It is worth to point out that the minimum is not zero and the maximum is not one due to the effect of the Fresnel coefficients [Eq. (7)]. Moreover, the height of maxima and minima depend on NA.

**Figure 3:**
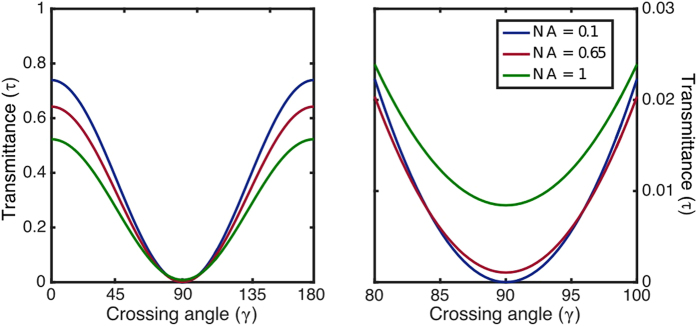
Transmittance when the field crosses successively two O-type polarisers forming a *γ* angle for NA = 0.1, 0.65, 1.

Another interesting issue is the analysis of the behaviour of a field that successively crosses two O-type polarisers whose axes form an angle *γ*. Let **E**_*pp*_ be the field after the second polariser. This distribution can be derived using Eqs (4) and (8). After some algebra, **E**_*pp*_ reads


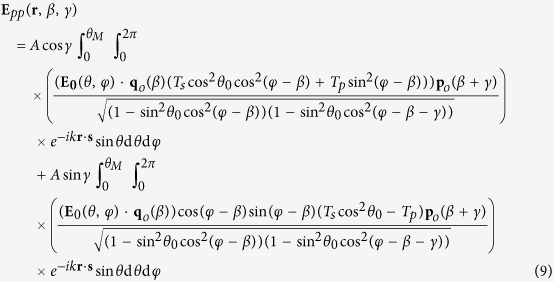


where *T*_*s*_ = *t*′_*s*_*t*_*s*_ and *T*_*p*_ = *t*′_*p*_*t*_*p*_. Note that for *γ* = 0 (two parallel polarisers), **E**_*pp*_(**r**, *β*, 0) ≠ **E**_*p*_(**r**, *β*). Therefore, an ideal polariser does not behaves as a projector for highly focused fields. Again, this fact is a consequence of the presence of the Fresnel coefficients [see Eq. (7)]. Nevertheless, for systems with small NA, *T*_*s*_ ≈ *T*_*p*_ ≈ 1 then 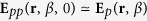 and the usual projector character of linear polarisers is recovered. On the other hand, if *γ* = *π*/2 (two crossed polarisers) the output field is not zero and therefore the usual Malus’ law does not apply. On top of that, if the influence of the Fresnel coefficients is neglected, *T*_*s*_ ≈ *T*_*p*_ ≈ 1, **E**_*pp*_(**r**, *β, π*/2) = 0, the usual result for normal incidence is recovered. Figure 3a shows the behaviour of the transmittance after crossing two polarisers as a function of *γ* following Eq. (9). Figure 3b displays a zoom of Fig. 3a around *γ* = 90°. In particular, for *γ* = 90°, **E**_*pp*_ ≠ 0 and the value of the minima increases with NA. Figures 2 and 3 can be reproduced with the codes provided in the Supplementary Information Section.

## Experimental results

**Figure 4:**
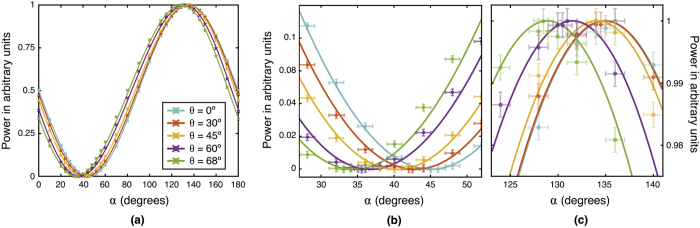
(**a**) Measured and calculated integrated irradiances of **E**_*p*_ as a function of polariser angle *α* for different angles of incidence *θ*. (**b**) and (**c**) display a zoom in the minima and maxima areas, respectively. Errors bars indicates the precision in the data acquisition for both angular and irradiance measurements.

In order to verify the theoretical approach presented in the previous section, several experiments have been conducted. First, we analyse the behaviour of a plane wave that impinges on a O-type polariser with a certain angle *θ*. The optical setup is presented in subsection ‘Experimental Set-ups’ (see below). According to Eq. (4), the emerging beam is


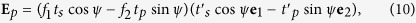


and the irradiance of this field reads


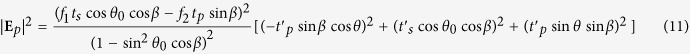


when the system is illuminated with a linearly polarised plane wave with *f*_1_ = *f*_2_ = 1 and *φ* = 0. A power-meter measures the total integrated irradiance of the transmitted beam **E**_*p*_. Figure 4 shows the total irradiance of this beam as a function of the polarization angle *α* = *β* + *π*/2 for different values of the incidence angle *θ*. Solid lines indicate the calculated values using Eq. (11) whereas the dots represent experimental data. Figures 4b and c display a zoom of the plots in the maxima and minima areas. The curves display a clear shift for the position of maxima and minima as a function of the angle of incidence *θ*, compatible with Eq. (11). Experimental data for generating Fig. 4 is available in the Supplementary Information section.

**Figure 5:**
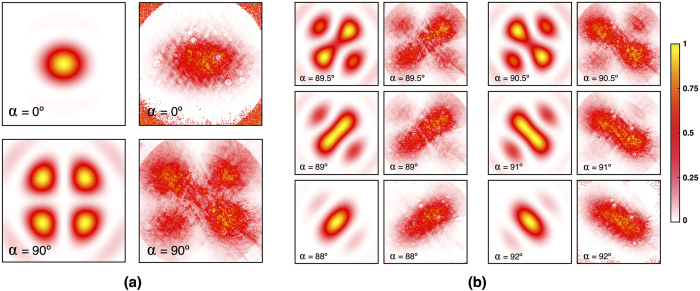
Focused fields after the polariser: (**a**) *α* = 0° and *α* = 90°. Experimental results are presented on the right whereas the corresponding numerical distributions are shown on the left. (**b**) Experimental and numerical irradiances for polarization angles close to *α* = 90°.

A second experiment has been carried out. In this case, a horizontal-linearly polarised plane wave (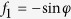 and 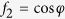) is focused using a microscope objective lens with NA = 0.65. The beam passes through a O-type linear polariser located at the focal plane and then a CCD camera records the irradiance. Figure 5a shows the irradiance distributions recorded by the camera for two positions of the polariser, namely *α* = 0° and *α* = 90°. For comparison purposes, |**E**_*p*_(**r**, *α*)|^2^ has been computed using Eq. (8) and they are depicted in the left column. Figure 5b shows experimental and numerical irradiances |**E**_*p*_(**r**, *α*)|^2^ for values of *α* close to 90°. Note that beam irradiance displays a cross-like distribution for *α* = 90° but this shape disappears when the polariser is rotated just a few degrees. Experimental images are noisy because the energy of the irradiance that reaches the camera is very low for *α* ≈ 90°, as described in Fig. 2c.

## Discussion

As explained above, the properties of a highly focused field after a linear polariser are substantially different to the characteristics of a wave propagating in the paraxial regime. More specifically, the longitudinal component is modified and polarisers do not behave as projectors when interact with focused fields; therefore, the usual Malus’ law does not strictly apply. We used a model for linear polarisers based on uniaxial anisotropic plates where multiple internal reflections are neglected. According to this approach, the effect of a polariser on the focal plane of a high NA objective lens can be described by using the plane waves spectrum representation of electromagnetic fields. It is worth to point out that the usual projector character of the polariser is recovered in the paraxial approach when the influence of the Fresnel coefficients is neglected. We conducted several experiments for verifying our theoretical framework. First, we analysed the behaviour of a paraxial beams impinging the polariser with a certain angle and then, linearly polarised plane waves were focused using a microscope objective. The irradiance distribution and the total integrated irradiance were measured. Results showed a good agreement between theoretical and experimental data.

## Methods

### Theoretical Background: Polarisers as uniaxial anisotropic media

**Figure 6:**
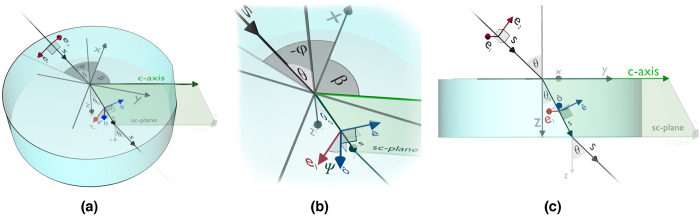
Coordinate system and geometrical magnitudes. (**a**) General view. (**b**) View of **e**_1_, 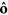, 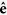 and 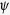. (**c**) Profile view of the *z*- and *c*- plane.

Polarisers and retarder plates have been described as a uniaxial anisotropic plane-parallel media of thickness *L* with the optical axis (*c*-axis) parallel to the plate surfaces^21^,^22^. This description provides an appropriate mathematical framework for explaining the behaviour of light when interacts with these devices. The coordinate system is selected in such a way that the *z*-axis is orthogonal to the plate surface, being *β* the angle between the *c*- and the *x*-axes. Accordingly, the direction of the *c*-axis is described by vector 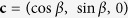. Figure 6 describes the geometrical magnitudes involved in the problem. In particular, angle Ψ [see Eq. (6)] is defined by 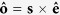 and ***e***_1_, where 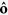 and 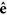 are the ordinary and extraordinary unit vectors.

Let us consider a plane wave with a wave-front vector **s** impinging an anisotropic plate from subspace *z* < 0; *θ* and *ϕ* are the polar and azimuthal angles respectively. **E**_*i*_ and **E**_*p*_(*β*) are the electric fields of the incident and the transmitted wave-fronts respectively:


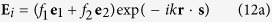



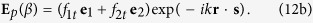


Provided that multiple internal reflections can be neglected, components *f*_1_, *f*_2_, *f*_1*t*_ and *f*_2*t*_, are determined by means of the so called Dynamical Matrices^18^,^19^,^20^. Derivation of these parameters can be carried out under the small-birefringence approximation, i.e. 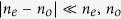, where *n*_*o*_ and *n*_*e*_ are the ordinary and extraordinary indices respectively. This approximation is valid for a large number of birefringent materials and devices including polarisers and liquid crystals^21^,^22^. If this approximation holds, the relationship between incident and transmitted electric fields reads


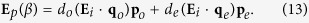


Quantities *d*_*o*_ and *d*_*e*_ are given by 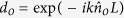 and 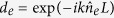, where 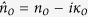 and 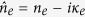 are the ordinary and extraordinary complex refractive indices.

From a physical point of view, Eq. (13) decomposes the incident beam into a linear combination of the normal modes of propagation (ordinary and extraordinary) along with the Fresnel transmission on the two surfaces of the plate. Vectors **p**_*o*_ and **p**_*e*_ fulfil 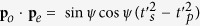 and therefore, they are not orthogonal unless the crystal axis direction ***c*** is parallel or perpendicular to the incident plane. For normal incidence (*θ* = 0), *t*_*s*_ = *t*_*p*_ and *t*′_*s*_ = *t*′_*p*_; therefore, vectors **p**_*o*_ and **q**_*o*_ are parallel to the unitary vector 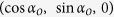, with *α*_*o*_ = *β* + *π*/2; **p**_*e*_ and **q**_*e*_ are parallel to vector 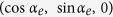, with *α*_*e*_ = *β*. It should be noted that s- or p- polarization is not preserved, unless *ψ* = 0 (for the ordinary wave) or *ψ* = *π*/2 (for the extraordinary wave).

Light propagating through a uniaxial polarisers suffers a strong attenuation of one of the propagation modes within the bulk of the polariser. Accordingly, polarisers can be classified into two types:

- O-type polarisers transmit ordinary waves and attenuates extraordinary ones, i.e. 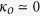 and *κ*_*e*_ > 0 and in this case, 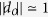 and 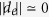. Thus, for this type of polarisers, the so-called polarization angle *α* is orthogonal to the *c*-axis, i.e. *α* = *α*_*o*_ = *β* + *π*/2.
- E-type polarisers transmit extraordinary waves and attenuates ordinary ones, i.e. *κ*_*o*_ > 0 and 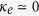. Equivalently, 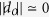 and 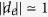. Thus, for this type of polarisers, the so-called polarization angle *α* is parallel to the *c*-axis, i.e. *α* = *α*_*e*_ = *β*.

Then, the transmitted field ***E***_*p*_ [Eq. (13)] for the O- and E-type polarisers becomes


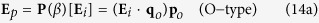



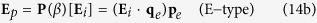


The plane wave after the polariser is linearly polarised along ***p***_*o*_ or ***p***_*e*_ and due to the Fresnel coefficients in Eq. (5), an ideal polariser does not behave as a projector operator even for normal incidence (*θ* ≈ *θ*_0_). Nevertheless, if the contribution of the Fresnel coefficients is not taken into account, Eqs (14a) and (14b) are in agreement with the model used in refs 1 and 2.

The polarisers used in the experiments are produced by stretching polymer polyvinyl alcohol. According to^21^, this technique produces an alignment of the anisometric molecules in such a way that the extinction direction is parallel to the molecular structures. In the present model, the polarisers are considered to be O-type because the *c*-axis is parallel to the absorption axis. Moreover, the polarization direction 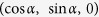 is orthogonal to the *c*-axis, i.e. *α* = *β* + *π*/2.

### Experimental set-ups

**Figure 7:**
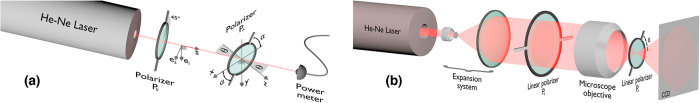
Optical set-ups.

Two different optical set-ups have been developed: The first one is used to analyse the behaviour of a linearly polarised plane wave that impinges on a polariser at a certain angle of incidence *θ* (see Fig. 7a). The light source is a conventional non-polarised He-Ne laser; the beam is polarised using linear polariser P_0_ (*α* = 45°) placed at the direction normal to the propagation direction. A second polariser P_*A*_ is mounted on a goniometer and thus, precise measurements of incidence angle *θ* can be carried out. Finally, a power-meter is used to measure the integrated irradiance transmitted by polariser P_*A*_. Several values of the integrated irradiance have been recorded as a function of *α* (for polarizer P_*A*_). More measurements were carried around *α* = 40° and *α* = 135° because more resolution is required. The angle of incidence was set to *θ* = 0°, 30°, 45°, 60°, 68° with a precision of ±1°. The results are presented in the Fig. 4 where the error bars show the precision of the acquired data. The precision of the polarization angle *α* is ±1° whereas the error associated to the total integrated irradiance depends on the scale used in the power-meter.

The second system (depicted in Fig. 7b) uses also a He-Ne laser as a light source. The beam is expanded and crosses polariser P_0_; the field is focused using a microscope objective with a NA = 0.65. Despite the fact the NA of the objective lens is not very high, the recorded beams show a distinctive behaviour when compared with paraxial beams. Moreover, if a objective lens with a higher NA was used, the size of the focused field would be so small that the details of the field distribution could not be detected by the camera. Analyser P_*A*_ is set at the focal plane of the objective lens and afterwards, a CCD camera records the irradiance distribution transmitted by polariser P_*A*_. The precision for angle *α* is around ±0.5°. The integrated irradiance shown in Fig. 2b and c is obtained with a power-meter after polariser P_*A*_.

## Additional Information

**How to cite this article:** Martínez-Herrero, R. *et al*. Polarisers in the focal domain: Theoretical model and experimental validation. *Sci. Rep.* **7**, 42122; doi: 10.1038/srep42122 (2017).

**Publisher's note:** Springer Nature remains neutral with regard to jurisdictional claims in published maps and institutional affiliations.

## Supplementary Material

Supplementary Information

Main Matlab Script

Matlab function

Expermental data
