# Supplementary material for: Polarisers in the focal domain: Theoretical model and experimental validation
Source: Sci Rep. 2017 Feb 13;7:42122. doi: 10.1038/srep42122 (PMC5304165; doi:10.1038/srep42122)
Supplement: Supplementary Information [file srep42122-s1.pdf]

README FIRST

Supplementary information files corresponding to the manuscript 'Polarisers in the focal domain: Theoretical model and experimental validation' by R. Martínez-Herrero, D. Maluenda, I. Juvells and A. Carnicer

FIGURES_SciRep.m: a MatLab script for generating Figures 2, 3 and 5.

RWinTrapz.m: a MatLab function that computes the Richards-Wolf integral. RWinTrapz()is called several times by the 'FIGURES_SciRep.m' script.

ExperimentalData_Fig4.xls: a MS Excel table containing the experimental data acquired and depicted in Fig. 4.
